# Supplementary material for: Cytokine response during non-cerebral and cerebral malaria: evidence of a failure to control inflammation as a cause of death in African adults
Source: PeerJ. 2016 May 2;4:e1965. doi: 10.7717/peerj.1965 (PMC4860323; doi:10.7717/peerj.1965)
Supplement: Supplemental Information 5 [file peerj-04-1965-s006.pdf]

```
NPAR TESTS
  /M-W= EGF Eotaxin GCSF GMCSF IFNa2 IFNg IL10 IL12p40 IL12p70 IL13 IL15 IL17
A IL1RA IL1a IL1b IL2 IL3 IL4 IL5 IL6 IL7 IL8 IP10 MCP1
    MIP1a MIP1b TNFa TNFb VEGF BY Kidney(0 1)
  /MISSING ANALYSIS.
```

NPar Tests

Notes

	Output Created	01-Mar-2016 19:12:16
	Comments	
Input	Data	C:\Users\user\Desktop\YD FROM NOV-28\Hp and malaria\Cytokines IPDk\Stats Cytokine\NCM + CM.sav
	Active Dataset	DataSet2
	Filter	Status = 3 (FILTER)
	Weight	<none>
	Split File	<none>
	N of Rows in Working Data File	27
Missing Value Handling	Definition of Missing	User-defined missing values are treated as missing.
	Cases Used	Statistics for each test are based on all cases with valid data for the variable(s) used in that test.
	Syntax	NPAR TESTS /M-W= EGF Eotaxin GCSF GMCSF IFNa2 IFNg IL10 IL12p40 IL12p70 IL13 IL15 IL17A IL1RA IL1a IL1b IL2 IL3 IL4 IL5 IL6 IL7 IL8 IP10 MCP1 MIP1a MIP1b TNFa TNFb VEGF BY Kidney(0 1) /MISSING ANALYSIS.
Resources	Processor Time	0:00:00.031
	Elapsed Time	0:00:00.032
	Number of Cases Allowed[a]	22469

a. Based on availability of workspace memory.

```
[DataSet2] C:\Users\user\Desktop\YD FROM NOV-28\Hp and malaria\Cytokines IPDk
\Stats Cytokine\NCM + CM.sav
```

Mann-Whitney Test

Ranks

	Kidney failure	N	Mean Rank	Sum of Ranks
EGF	No	14	14.54	203.50
	Yes	13	13.42	174.50
	Total	27		
Eotaxin	No	14	10.14	142.00
	Yes	13	18.15	236.00
	Total	27		
G-CSF	No	14	10.25	143.50
	Yes	13	18.04	234.50
	Total	27		
GM-CSF	No	14	8.64	121.00
	Yes	13	19.77	257.00
	Total	27		
IFN-alpha2	No	14	10.07	141.00
	Yes	13	18.23	237.00
	Total	27		
IFN-gamma	No	14	10.04	140.50
	Yes	13	18.27	237.50
	Total	27		
IL-10	No	14	10.57	148.00
	Yes	13	17.69	230.00
	Total	27		
IL-12p40	No	14	13.39	187.50
	Yes	13	14.65	190.50
	Total	27		
IL-12p70	No	14	10.68	149.50
	Yes	13	17.58	228.50
	Total	27		
IL-13	No	14	13.50	189.00
	Yes	13	14.54	189.00
	Total	27		
IL-15	No	14	9.43	132.00
	Yes	13	18.92	246.00
	Total	27		
IL-17A	No	14	11.00	154.00
	Yes	13	17.23	224.00
	Total	27		
IL-1RA	No	14	11.07	155.00
	Yes	13	17.15	223.00

Ranks

	Kidney failure	N	Mean Rank	Sum of Ranks
IL-1RA	Total	27		
IL-1alpha	No	14	9.61	134.50
	Yes	13	18.73	243.50
	Total	27		
IL-1beta	No	14	11.86	166.00
	Yes	13	16.31	212.00
	Total	27		
IL-2	No	14	12.39	173.50
	Yes	13	15.73	204.50
	Total	27		
IL-3	No	14	13.50	189.00
	Yes	13	14.54	189.00
	Total	27		
IL-4	No	14	13.93	195.00
	Yes	13	14.08	183.00
	Total	27		
IL-5	No	14	13.50	189.00
	Yes	13	14.54	189.00
	Total	27		
IL-6	No	14	11.21	157.00
	Yes	13	17.00	221.00
	Total	27		
IL-7	No	14	13.36	187.00
	Yes	13	14.69	191.00
	Total	27		
IL-8	No	14	12.00	168.00
	Yes	13	16.15	210.00
	Total	27		
IP-10	No	14	10.00	140.00
	Yes	13	18.31	238.00
	Total	27		
MCP-1	No	14	9.64	135.00
	Yes	13	18.69	243.00
	Total	27		
MIP-1alpha	No	14	12.21	171.00
	Yes	13	15.92	207.00
	Total	27		
MIP-1beta	No	14	12.50	175.00
	Yes	13	15.62	203.00
	Total	27		

Ranks

	Kidney failure	N	Mean Rank	Sum of Ranks
TNFalpha	No	14	9.93	139.00
	Yes	13	18.38	239.00
	Total	27		
TNFbeta	No	14	13.50	189.00
	Yes	13	14.54	189.00
	Total	27		
VEGF	No	14	11.96	167.50
	Yes	13	16.19	210.50
	Total	27		

Test Statistics[b]

	EGF	Eotaxin	G-CSF	GM-CSF	IFN-alpha2	IFN-gamma
Mann-Whitney U	83.500	37.000	38.500	16.000	36.000	35.500
Wilcoxon W	174.500	142.000	143.500	121.000	141.000	140.500
Z	-.364	-2.620	-2.548	-3.642	-2.675	-2.698
Asymp. Sig. (2-tailed)	.716	.009	.011	.000	.007	.007
Exact Sig. [2*(1-tailed Sig.)]	.720[a]	.008[a]	.009[a]	.000[a]	.007[a]	.006[a]

a. Not corrected for ties.

b. Grouping Variable: Kidney failure

Test Statistics[b]

	IL-10	IL-12p40	IL-12p70	IL-13	IL-15	IL-17A
Mann-Whitney U	43.000	82.500	44.500	84.000	27.000	49.000
Wilcoxon W	148.000	187.500	149.500	189.000	132.000	154.000
Z	-2.330	-.756	-2.269	-1.038	-3.165	-2.042
Asymp. Sig. (2-tailed)	.020	.450	.023	.299	.002	.041
Exact Sig. [2*(1-tailed Sig.)]	.019[a]	.685[a]	.022[a]	.756[a]	.001[a]	.043[a]

a. Not corrected for ties.

b. Grouping Variable: Kidney failure

Test Statistics[b]

	IL-1RA	IL-1alpha	IL-1beta	IL-2	IL-3	IL-4
Mann-Whitney U	50.000	29.500	61.000	68.500	84.000	90.000
Wilcoxon W	155.000	134.500	166.000	173.500	189.000	195.000
Z	-1.990	-3.023	-1.545	-1.611	-1.038	-.107
Asymp. Sig. (2-tailed)	.047	.002	.122	.107	.299	.915
Exact Sig. [2*(1-tailed Sig.)]	.048[a]	.002[a]	.155[a]	.280[a]	.756[a]	.981[a]

a. Not corrected for ties.

b. Grouping Variable: Kidney failure

Test Statistics[b]

	IL-5	IL-6	IL-7	IL-8	IP-10	MCP-1
Mann-Whitney U	84.000	52.000	82.000	63.000	35.000	30.000
Wilcoxon W	189.000	157.000	187.000	168.000	140.000	135.000
Z	-1.038	-1.893	-.449	-1.359	-2.717	-2.960
Asymp. Sig. (2-tailed)	.299	.058	.653	.174	.007	.003
Exact Sig. [2*(1-tailed Sig.)]	.756[a]	.061[a]	.685[a]	.185[a]	.006[a]	.002[a]

a. Not corrected for ties.

b. Grouping Variable: Kidney failure

Test Statistics[b]

	MIP-1alpha	MIP-1beta	TNFalpha	TNFbeta	VEGF
Mann-Whitney U	66.000	70.000	34.000	84.000	62.500
Wilcoxon W	171.000	175.000	139.000	189.000	167.500
Z	-1.220	-1.019	-2.766	-1.038	-1.387
Asymp. Sig. (2-tailed)	.223	.308	.006	.299	.166
Exact Sig. [2*(1-tailed Sig.)]	.239[a]	.325[a]	.005[a]	.756[a]	.169[a]

a. Not corrected for ties.

b. Grouping Variable: Kidney failure
